# Study on the mechanism of Danshen-Guizhi drug pair in the treatment of ovarian cancer based on network pharmacology and *in vitro* experiment

**DOI:** 10.7717/peerj.13148

**Published:** 2022-04-06

**Authors:** Chongzhen Qin, Menglin Wu, Xinru Wang, Wenda Zhang, Guangzhao Qi, Na-Yi Wu, Xiaoting Liu, Yaoyao Lu, Jingmin Zhang, Yuna Chai

**Affiliations:** 1 The First Affiliated Hospital of Zhengzhou University, Zhengzhou, China; 2 Hunan Cancer Hospital, The Affiliated Cancer Hospital of Xiangya School of Medicine, Central South University, Changsha, China; 3 The Second People’s Hospital of Hunan Province, Changsha, China

**Keywords:** Danshen, Guizhi, Core targets, Network pharmacology, Ovarian cancer, Molecular docking

## Abstract

Our study aims to explore the active components and mechanisms of the Danshen-Guizhi drug pair in treating ovarian cancer by network pharmacology and *in vitro* experiment. The “component-target-pathway” diagram of the Danshen-Guizhi drug pair was established by network pharmacology, and the effective active components, important targets as well as potential mechanisms of the Danshen-Guizhi drug pair were analyzed. The predicted results were verified by molecular docking and *in vitro* experiments. The main active components of the Danshen-Guizhi drug pair in the treatment of ovarian cancer are salviolone, luteolin, β-sitosterol and tanshinone IIA. The main core target is PTGS2. The pathways involved mainly include the cancer pathway, PI3K-Akt signaling pathway, and IL-17 signaling pathway. The molecular docking results showed that salviolone and tanshinone IIA had good binding ability to the target. The expression of PTGS2 mRNA and PGE2 in ovarian cells were significantly inhibited by salviolone. The mechanism of the Danshen-Guizhi drug pair in the treatment of ovarian cancer may be regulating cell proliferation, apoptosis and tumor immunity. This provides a theoretical basis for the clinical development and application of the Danshen-Guizhi drug pair.

## Introduction

Ovarian cancer is one of the most common gynecologic malignancies. Its incidence ranks third in female reproductive system malignant tumors, after cervical cancer and uterine cancer (Hunn & Rodriguez, 2012). The early symptoms of ovarian cancer are unobvious, and late symptoms are untypical, the recurrence rate and mortality of ovarian cancer is the first in all kinds of gynecological tumors (Gaona-Luviano, Medina-Gaona & Magana-Perez, 2020). The treatment of ovarian cancer is mainly the comprehensive application of surgery and chemotherapy. However, surgical treatment has relatively large physical damage to patients, and there will be recurrence or serious complications, and platinum drug resistance is also a major problem in the treatment of ovarian cancer (McMullen, Madariaga & Lheureux, 2020).

Many clinical studies have shown that both a single Chinese herbal medicine and Chinese herbal formula for cancer treatment has been proved effective (Mao et al., 2017). It is reported that traditional Chinese medicine for the treatment of ovarian cancer plays an anti-cancer role mainly by affecting the proliferation and apoptosis of cancer cells, generating of tumor vessel and inhibiting the drug resistance of platinum chemotherapeutic drugs (Sun et al., 2020). *Salvia miltiorrhiza* Bunge (Radix Salviae, Danshen) was firstly recorded in *Shennong’s Herbal Classics*, which is a popular component of many traditional Chinese medicines. Previous studies have shown that the effective active ingredients of Danshen play a role in the development of ovarian cancer (Zhou et al., 2020; Wang et al., 2020; Yang et al., 2018). The water-soluble components of Danshen can promote blood circulation and remove blood stasis, and fat-soluble components have anti-inflammatory, antibacterial, antioxidant as well as anti-tumor effects (Su et al., 2015). Tanshinone, the fat-soluble component of Danshen, has antiproliferative effects on ovarian cancer cells by promoting apoptosis and down-regulating cisplatin resistance genes (Jiao & Wen, 2011). *Neolitsea cassia* (L.) Kosterm (Cinnamomi Ramulus, Guizhi) was first found in Newly Compiled Materia Medica of the Tang Dynasty. It can sweat and relieve external syndromes, warm meridians, improve blood circulation, and relieve pain (Liu et al., 2020). Guizhi mainly contains cinnamic acid and cinnamic aldehyde, and can inhibit the growth of cancer cells (Reddy et al., 2016). The main active components of Danshen and Guizhi can play a synergistic effect, and improve the efficacy of the two drugs, with effect of promoting blood circulation, dredging collaterals, tonifying *qi* (vital energy) and nourishing heart. Traditional Chinese medicine believes that the formation of ovarian cancer is due to blood stasis, the pathological mechanism is mainly the disharmony of *qi* and blood, *qi* stagnation and blood stasis, and then the formation of mass as proposed by Medical Required (Jiang & Lin, 2006). The Danshen-Guizhi drug pair can improve the blood circulation of patients, balance *qi* and blood, and remove blood stasis. However, its therapeutic mechanism has not been clearly clarified.

With the rapid development of bioinformatics, network pharmacology has become a new discipline, which mainly reveals the pharmacological mechanism of traditional Chinese medicine. The research and development mode has changed from the traditional “single target, high selective drug development, disease-single target-single drug” to “multi-direction pharmacology, drug action on biological networks, disease-multi-target-single drug”, which has promoted the modernization of traditional Chinese medicine (Zhang et al., 2013; Zhou et al., 2020). Network pharmacology is mainly to obtain disease-related targets from diseases by text mining, obtain effective components of drugs in traditional Chinese medicine compound for target prediction, construct drug-target-disease network, and then carry out network analysis to predict the disease mechanism (Li & Zhang, 2013). In this study, the network pharmacology method was used to explore the mechanism of the Danshen-Guizhi drug pair in the treatment of ovarian cancer with multi-component, multi-target and multi-pathway, to provide a theoretical basis for the further development and utilization of the Danshen-Guizhi drug pair in clinical practice.

## Materials and Methods

### Screening of effective chemical component and collecting targets of Danshen-Guizhi drug pair

The main effective components of the Danshen-Guizhi drug pair were collected from the TCMSP (https://tcmspw.com/tcmsp.php). All components of the drug pair were screened by TCMSP based on absorption, distribution, metabolism, and excretion (ADME), including oral bioavailability (OB), drug similarity (DL), and lipids-water partition coefficient (AlogP) (Lee et al., 2019). OB is the percentage of oral drugs absorbed into systemic circulation, DL represents the similarity between the active ingredients of Traditional Chinese medicine and drugs, AlogP is one of the binding forces between drugs and receptors, and the occurrence of drug pharmacokinetic process is related to the hydrophobicity of drugs (Liu et al., 2013). All components of the Danshen-Guizhi drug pair were screened under the following conditions: oral bioavailability (OB) ≥30%, drug-like property (DL) ≥0.18, and lipid-water partition coefficient (AlogP) ≤5 (Liu et al., 2016). The main active compounds of the Danshen-Guizhi drug pair and their respective protein targets were obtained, the collected targets were transformed into gene names in the Uniprot protein database (https://www.uniprot.org/uploadlists/), and invalid genes were deleted.

### Acquisition of disease-related targets

Enter “Ovarian Cancer” in Genecards database (https://www.genecards.org/) and OMIM database (https://omim.org/) to search for potential therapeutic targets of ovarian cancer.

### Construction of PPI network and screening of core targets

The ovarian cancer-related gene targets obtained in Genecards were crossed with the drug targets collected in TCMSP to obtain the potential targets for drug treatment of ovarian cancer, and the VENNY diagram was plotted. Then the intersection targets were imported into the STRING (https://www.string-db.org/) database to restrict the species to human (“*Homo sapiens*”), and the protein-protein interaction network function enrichment analysis was carried out to obtain the PPI network. The results were topologically analyzed by Cytoscape3.8.0 software plug-in for potential therapeutic targets, and PPI network diagram of potential therapeutic targets of the Danshen-Guizhi drug pair for ovarian cancer was constructed.

In order to explore the mechanism of the Danshen-Guizhi drug pair in the treatment of ovarian cancer, the obtained PPI of disease and drug were combined with the intersection network in Cytoscape3.8.0 software, and then the topology network was analyzed by Cytoscape3.8.0 software plug-in to screen the core targets.

### Enrichment analysis of Danshen-Guizhi drug pair in the treatment of ovarian cancer

The Matescap database (http://metascape.org/gp/index.html#/main/step1) is a powerful online tool for gene enrichment analysis, which can be used for gene annotation and analysis resources. The potential targets of the Danshen-Guizhi drug pair in the treatment of ovarian cancer were input into the Matescape database, and the species was limited to “*H. sapiens*”. *P* < 0.01 was set for enrichment analysis of GO molecular function, cell composition and KEGG pathway.

### Danshen-Guizhi drug pair “component-target-pathway” network construction

The network diagrams of active components, targets and pathways of drugs were drawn by Cytoscape 3.8.0 software. The network topology parameters of active components and targets of drugs were analyzed by CytoScape 3.8.0 plug-in CytoNCA. The core targets and main active components of drugs were judged according to the network topology parameters of Degree, Betweenness and Closeness.

### Molecular docking verification

The main components corresponding to the target protein were searched from the TCMSP database (https://tcmspw.com/tcmsp.php), downloaded and saved as Mol2 format. The “component-target-pathway” network of the Danshen-Guizhi drug pair was analyzed, and the core targets with degree greater than 20 were selected for docking. The corresponding PDB ID of the core targets was found in the PBD (http://www1.rcsb.org/) database and saved as pdb format. Then the target was dehydrated and hydrogenated to optimize ligand in Pymol (Ren et al., 2020). Finally, the molecular docking was performed by AutoDock software to calculate the binding ability of the main active components of the Danshen-Guizhi drug pair to the core targets.

### MTS assay

SKOV3 cells and A2780 cells were seeded in 96-well plates at 100 μL/well (about 1 × 10^4^), cultured at 37 °C in 5% CO_2_ incubator for 24 h. Luteolin, salviolone, tanshinone IIA and β-sitosterol were added at concentrations 0, 10 or 20 μm. Luteolin, β-sitosterol and tanshinone IIA were obtained from Sigma (USA) and salviolone was obtained from Tianjin Wanxiang Hengyuan Technology Co., LTD. After incubation for 2 h, the cell vitality was detected by MTS kit (G3582; Promega, Madison, WI, USA), and the optical density was detected at 450 nm.

### Detection of PTGS2 mRNA expression by qRT-PCR

Molecular docking results showed that the docking results of salvianone with seven main targets were the best. In order to further explore the role of salviolone in ovarian cancer, we detected the effect of salviolone on PTGS2 mRNA expression in ovarian cells. SKOV3 cells and A2780 cells were seeded in a 6-well plate and added with 0 and 20 μm of salviolone for 24 h. Cells were collected, total RNA was extracted, and PTGS2 mRNA was quantitatively detected. Data were represented as the Mean ± SD of three independent experiments.

### Detection of PGE2 expression by ELISA

SKOV3 cells were seeded in 6-well plates at 1 × 10^5^cells per well. Cells were incubated in serum-free medium. The cells were incubated with 0, 20 μm salviolone for 2 h. The cell supernatant was collected and PGE2 was measured by ELISA kit (ab133021; Abcam, Cambridge, UK). The absorbance (OD) of the sample was measured at 415 nm. The average value was calculated by three repeated experiments.

### Statistical analysis

SPSS Statistics 20.0 and GraphPad Prism v8.0.2 were used for statistical analysis. The data are expressed as Mean ± SD. Student’s *t*-test was used to analyze the difference in effect of luteolin, salviolone, β-sitosterol as well as tanshinone IIA on ovarian cancer cells, compared with the control group, and *P* < 0.05 was considered to be statistically significant.

## Results

### Obtaining active components and potential targets of Danshen-Guizhi drug pair

Through searching the TCMSP database, 202 effective active ingredients of Danshen and 220 effective active ingredients of Guizhi were obtained. According to ADME, 54 effective active ingredients of Danshen and six effective active ingredients of Guizhi were obtained. A total of 916 targets of effective components of Danshen and 74 targets of Guizhi were obtained. After deleting duplicate targets, 60 effective active ingredients and 136 targets were obtained, as shown in Table 1. The collected targets were transformed into genes in the Uniprot database.

**Table 1:** Major ingredients of the drug.

Medicine	MOL ID	Sign	Main active ingredient	OB(%)	DL(%)
Danshen	MOL001601	DS1	1,2,5,6-tetrahydrotanshinone	38.75	0.36
	MOL001942	DS2	isoimperatorin	45.46	0.23
	MOL002222	DS3	sugiol	36.11	0.28
	MOL002651	DS4	Dehydrotanshinone II a	43.76	0.4
	MOL002776	DS5	Baicalin	40.12	0.75
	MOL000569	DS6	digallate	61.85	0.26
	MOL000006	DS7	luteolin	36.16	0.25
	MOL007036	DS8	5,6-dihydroxy-7-isopropyl-1,1-dimethyl-2,3-dihydrophenanthren-4-one	33.77	0.29
	MOL007041	DS9	2-isopropyl-8-methylphenanthrene-3,4-dione	40.86	0.23
	MOL007045	DS10	3α-hydroxytanshinoneIIa	44.93	0.44
	MOL007048	DS11	(E)-3-[2-(3,4-dihydroxyphenyl)-7-hydroxy-benzofuran-4-yl]acrylic acid	48.24	0.31
	MOL007049	DS12	4-methylenemiltirone	34.35	0.23
	MOL007050	DS13	2-(4-hydroxy-3-methoxyphenyl)-5-(3-hydroxypropyl)-7-methoxy-3-benzofurancarboxaldehyde	62.78	0.4
	MOL007058	DS14	formyltanshinone	73.44	0.42
	MOL007059	DS15	3-beta-Hydroxymethyllenetanshiquinone	32.16	0.41
	MOL007061	DS16	Methylenetanshinquinone	37.07	0.36
	MOL007063	DS17	przewalskin a	37.11	0.65
	MOL007064	DS18	przewalskin b	110.32	0.44
	MOL007068	DS19	Przewaquinone b	62.24	0.41
	MOL007069	DS20	przewaquinone c	55.74	0.4
	MOL007070	DS21	(6S,7R)-6,7-dihydroxy-1,6-dimethyl-8,9-dihydro-7H-naphtho[8,7-g] benzofuran-10,11-dione	41.31	0.45
	MOL007071	DS22	przewaquinone f	40.31	0.46
	MOL007077	DS23	sclareol	43.67	0.21
	MOL007079	DS24	tanshinaldehyde	52.47	0.45
	MOL007081	DS25	Danshenol b	57.95	0.56
	MOL007082	DS26	Danshenol a	56.97	0.52
	MOL007085	DS27	Salvilenone	30.38	0.38
	MOL007088	DS28	cryptotanshinone	52.34	0.4
	MOL007093	DS29	dan-shexinkum d	38.88	0.55
	MOL007094	DS30	danshenspiroketallactone	50.43	0.31
	MOL007098	DS31	deoxyneocryptotanshinone	49.4	0.29
	MOL007100	DS32	dihydrotanshinlactone	38.68	0.32
	MOL007101	DS33	dihydrotanshinone I	45.04	0.36
	MOL007105	DS34	epidanshenspiroketallactone	68.27	0.31
	MOL007108	DS35	isocryptotanshi-none	54.98	0.39
	MOL007111	DS36	Isotanshinone II	49.92	0.4
	MOL007119	DS37	miltionone I	49.68	0.32
	MOL007120	DS38	miltionone II	71.03	0.44
	MOL007121	DS39	miltipolone	36.56	0.37
	MOL007122	DS40	Miltirone	38.76	0.25
	MOL007124	DS41	neocryptotanshinone II	39.46	0.23
	MOL007125	DS42	neocryptotanshinone	52.49	0.32
	MOL007127	DS43	1-methyl-8,9-dihydro-7H-naphtho[5,6-g] benzofuran-6,10,11-trione	34.72	0.37
	MOL007130	DS44	prolithospermic acid	64.37	0.31
	MOL007132	DS45	(2R)-3-(3,4-dihydroxyphenyl)-2-[(Z)-3-(3,4-dihydroxyphenyl) acryloyl] oxy-propionic acid	109.38	0.35
	MOL007141	DS46	salvianolic acid g	45.56	0.61
	MOL007143	DS47	salvilenone I	32.43	0.23
	MOL007145	DS48	salviolone	31.72	0.24
	MOL007150	DS49	(6S)-6-hydroxy-1-methyl-6-methylol-8,9-dihydro-7H-naphtho[8,7-g] benzofuran-10,11-quinone	75.39	0.46
	MOL007151	DS50	Tanshindiol b	42.67	0.45
	MOL007152	DS51	Przewaquinone e	42.85	0.45
	MOL007154	DS52	tanshinone iia	49.89	0.4
	MOL007155	DS53	(6S)-6-(hydroxymethyl)-1,6-dimethyl-8,9-dihydro-7H-naphtho[8,7-g] benzofuran-10,11-dione	65.26	0.45
	MOL007156	DS54	tanshinone VI	45.64	0.3
Guizhi	MOL001736	GZ1	(-)-taxifolin	60.51	0.27
	MOL000358	GZ2	beta-sitosterol	36.91	0.75
	MOL000359	GZ3	sitosterol	36.91	0.75
	MOL000492	GZ4	(+)-catechin	54.83	0.24
	MOL000073	GZ5	ent-Epicatechin	48.96	0.24
	MOL004576	GZ6	taxifolin	57.84	0.27

### Acquisition of ovarian cancer-related targets

A total of 8,366 potential targets related to ovarian cancer were obtained by Genecards human gene database. According to the target targets with Score greater than the median, the potential targets for the treatment of ovarian cancer were determined. Finally, 1,119 therapeutic targets for ovarian cancer were obtained (Table S1).

### Construction and analysis of PPI network

**Figure 1:**
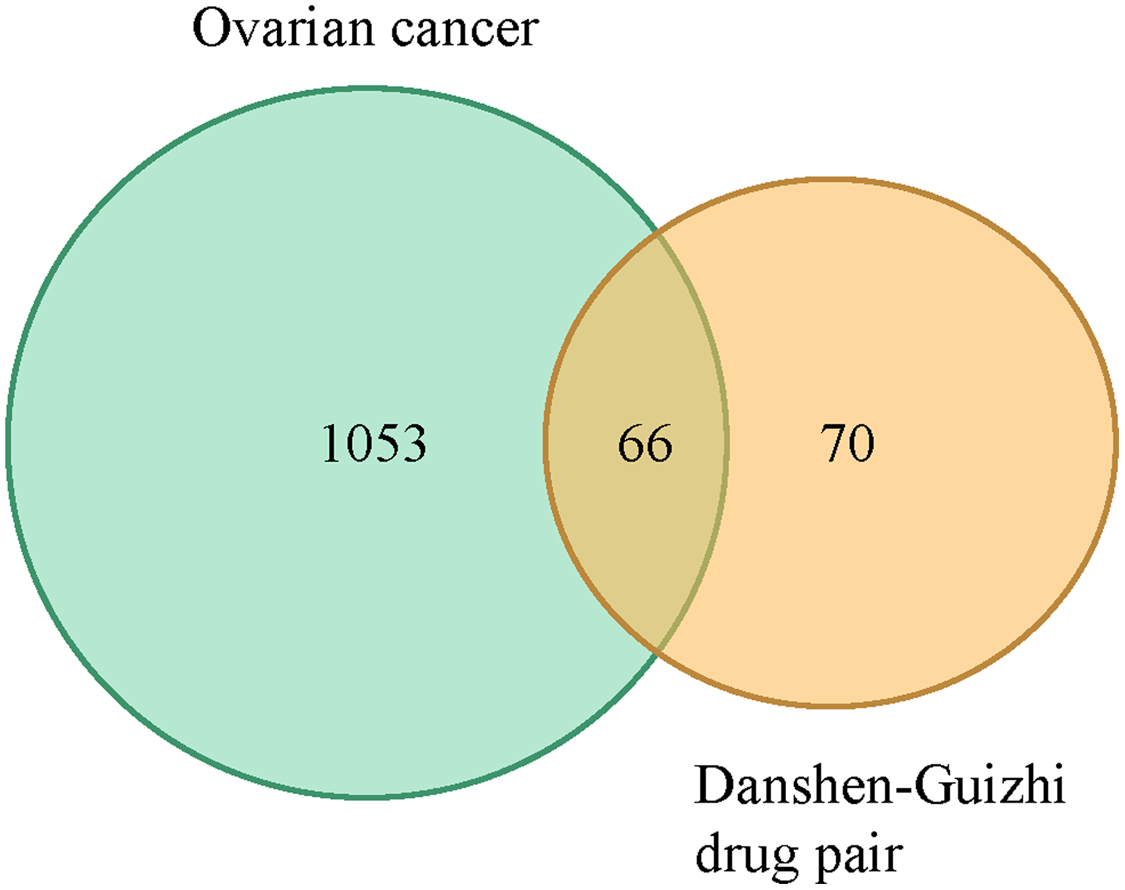
Venn diagram of active components of the Danshen-Guizhi drug pair and targets of ovarian cancer.

**Figure 2:**
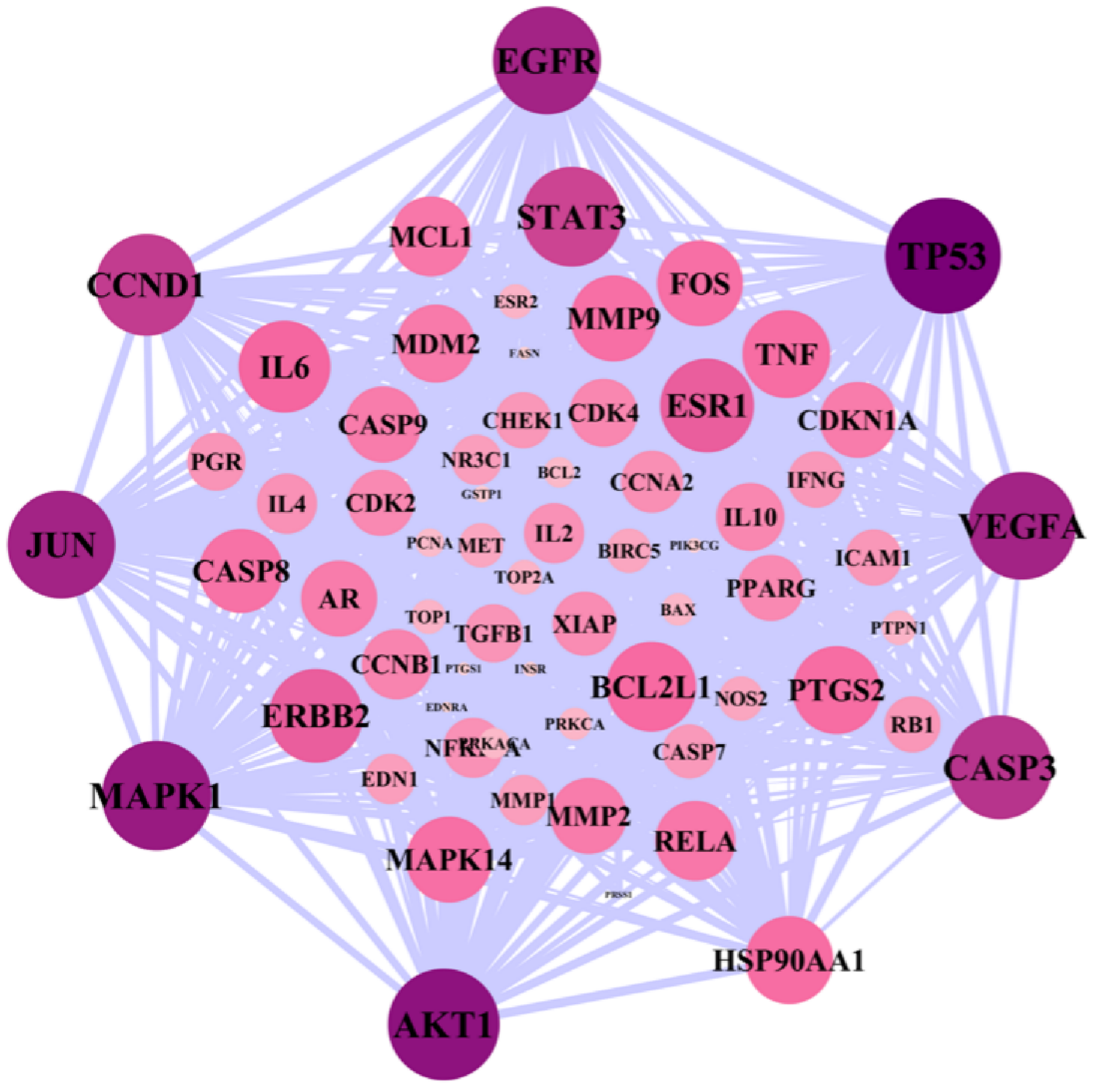
PPI network diagram. The greater the degree value, the larger the node and the darker the color.

A total of 119 ovarian cancer-related gene targets obtained in Genecards were crossed with 136 drug targets, and 66 potential targets for drug treatment of ovarian cancer were obtained. VENNY map was drawn, as shown in Fig. 1. The PPI network was constructed by STRING software for 66 targets. The network nodes represent proteins, and the edges represent the combination of proteins and proteins. The number of nodes is 66,1109. Cytoscape 3.8.0 software was used to construct the PPI network diagram of potential targets for the treatment of ovarian cancer by the Danshen-Guizhi drug pair, and the depth of node color as well as the size of node were related to the degree value. Gene targets can be screened from the color of node size: PTGS1, TP53, PTGS2, AKT1, MAPK1, JUN, AR, EGFR, ESR1, etc. These can be considered to be the core targets in the target interaction network of the Danshen-Guizhi drug pair for the treatment of ovarian cancer (Fig. 2).

**Figure 3:**
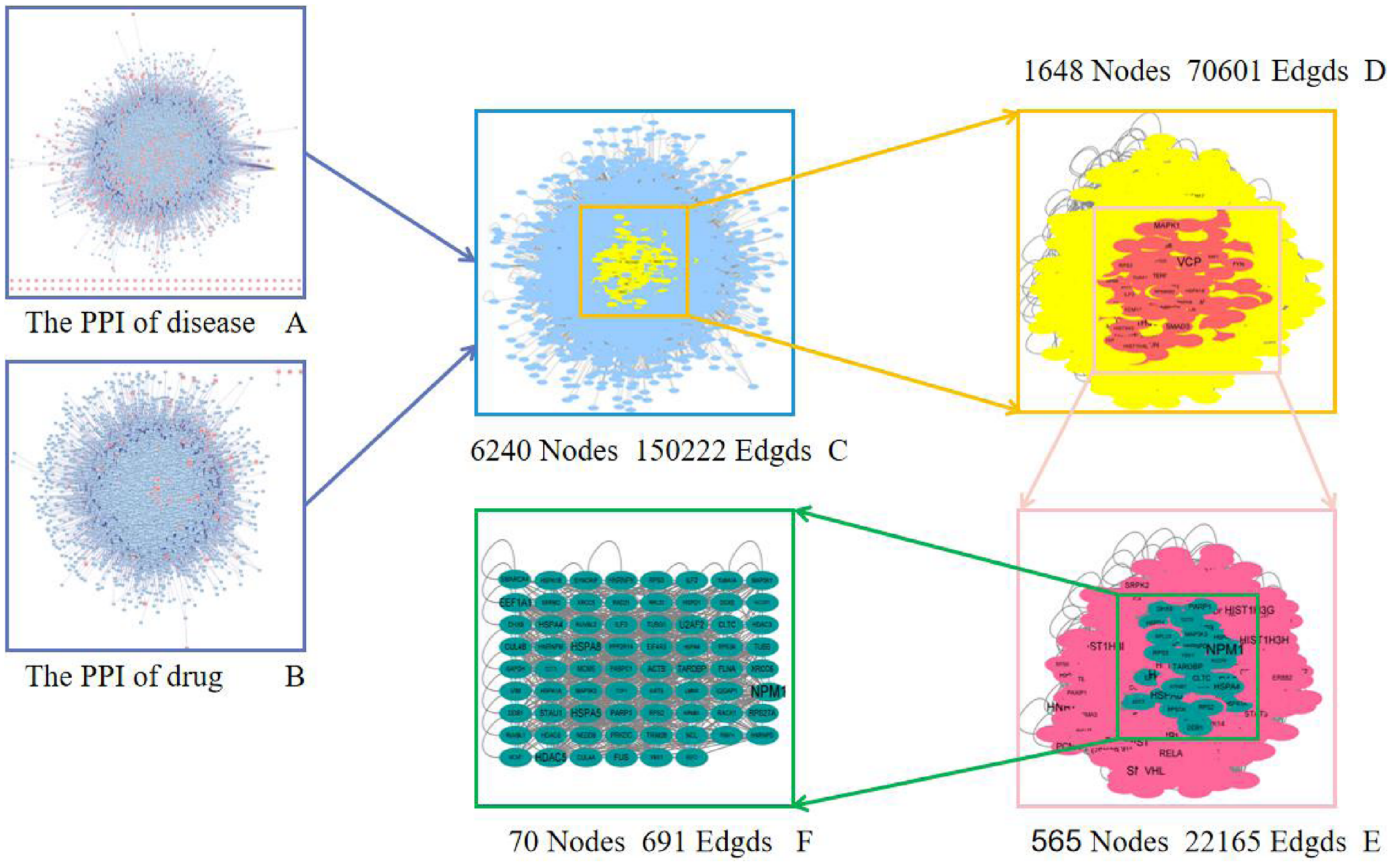
Screening of a core PPI network for Danshen-Guizhi drug pair in the treatment of ovarian cancer.

In order to reveal the pharmacological mechanism of the Danshen-Guizhi drug pair in the treatment of ovarian cancer, the PPI of disease (Fig. 3A) and drug (Fig. 3B) were combined to obtain the intersection network (Fig. 3C). The degree value of all nodes was calculated according to the median of two times. All nodes with the degree value greater than two times the median were selected to extract the network, and the Hithubs1 network (Fig. 3D) was obtained. The median and each attribute value of nodes in the Hithubs network were calculated. All nodes with the degree value greater than the median were selected to extract the network again, and the Hithubs2 network (Fig. 3E) was obtained. Finally, 70 key targets (Fig. 3F) were obtained.

### Enrichment analysis of potential targets for the treatment of ovarian cancer by Danshen-Guizhi drug pair

**Figure 4:**
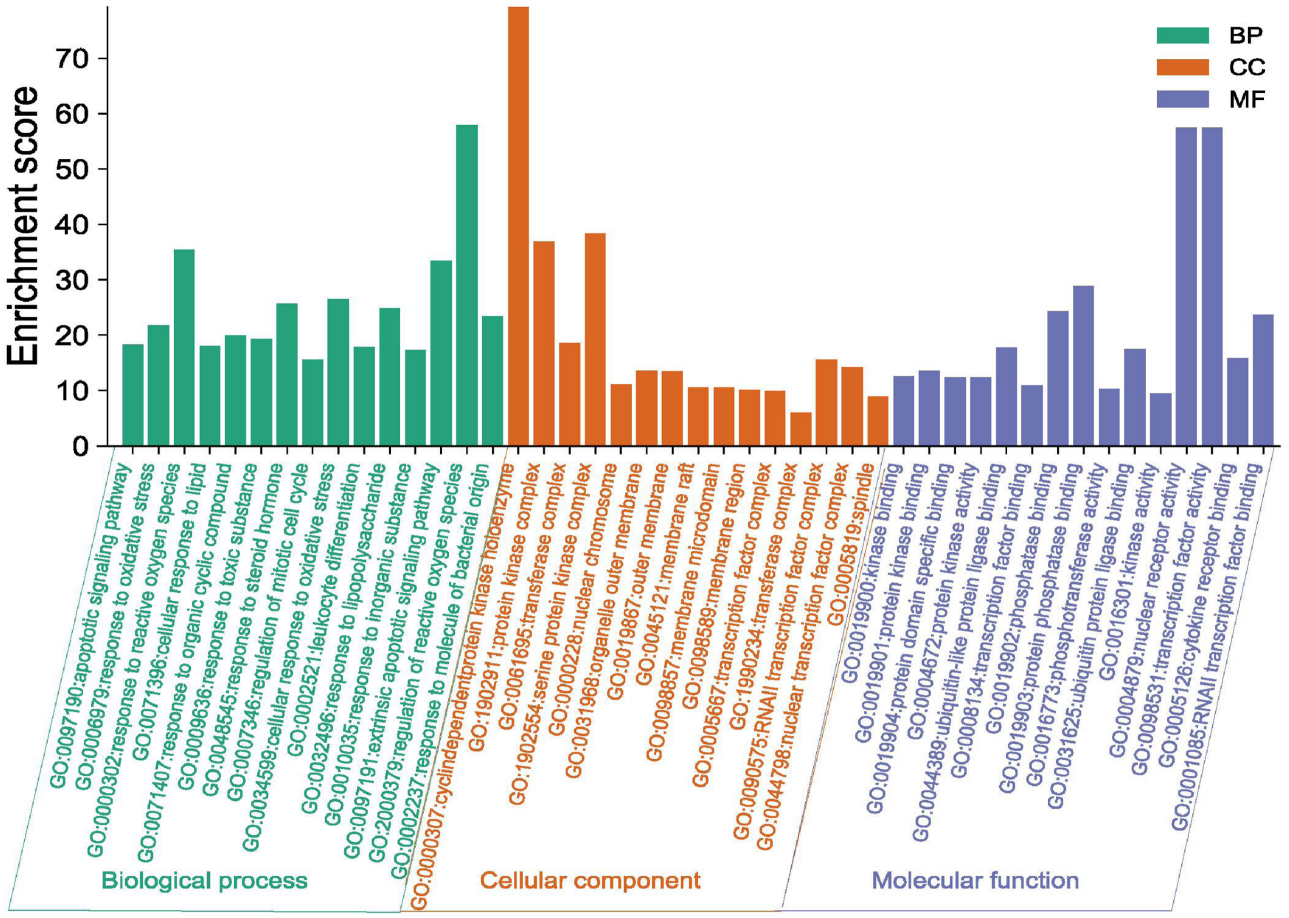
GO enrichment analysis of the Danshen-Guizhi drug pair on key targets for the treatment of ovarian cancer.

The potential targets of Danshen-Guizhi drug pair in the treatment of ovarian cancer were input into Matescape database for analysis, and the limited type was “*H. sapiens*”. The personalized analysis was carried out, and the *P* < 0.01 was set. The enrichment analysis of potential targets of the Danshen-Guizhi drug pair in the treatment of ovarian cancer was obtained, as shown in Fig. 4. The biological processes of GO enrichment include: apoptotic signaling pathway, response to oxidative stress, response to reactive oxygen species, regulation of mitotic cell cycle, cellular response to lipid, response to organic cyclic compound and response to steroid hormone.

**Figure 5:**
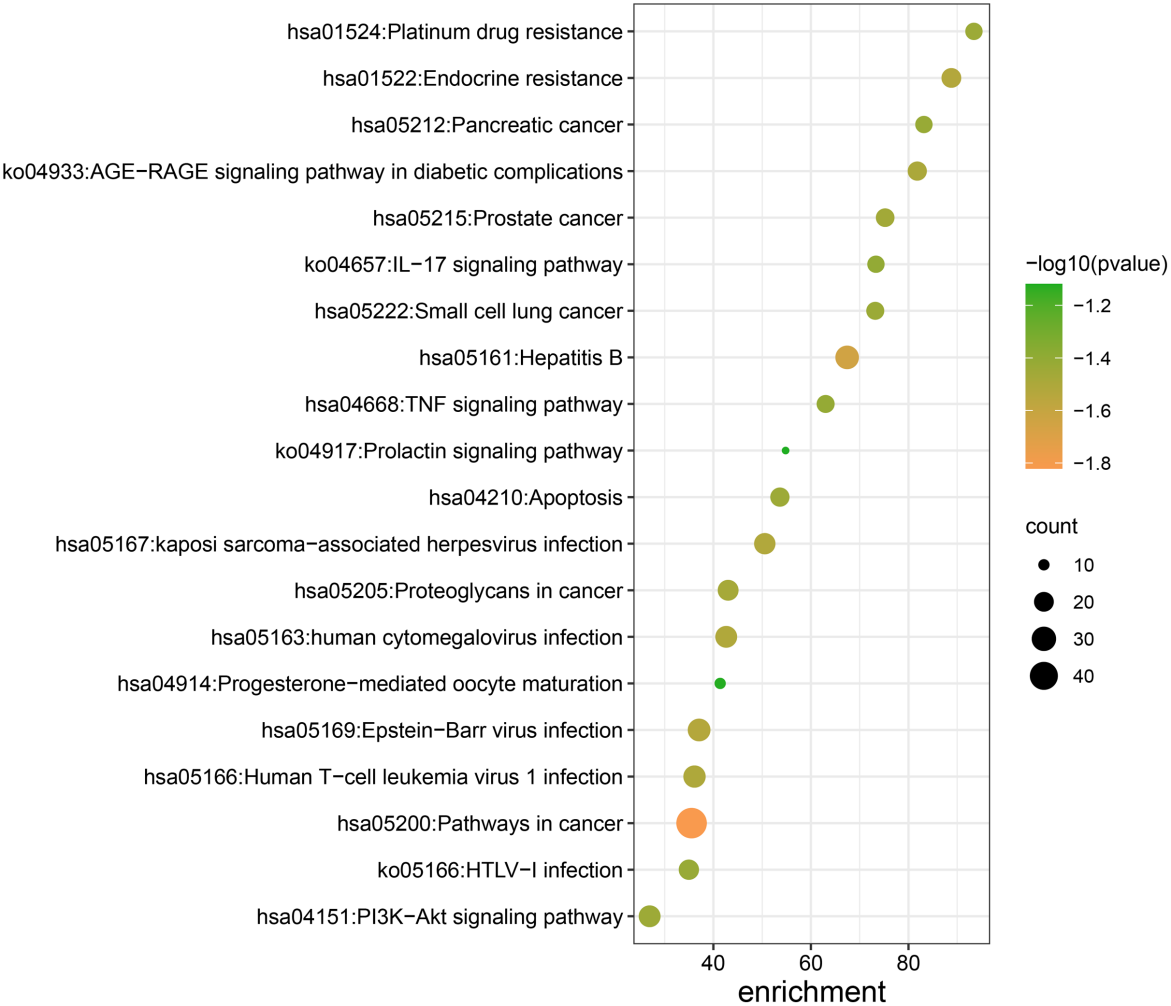
KEGG pathway enrichment analysis of Danshen-Guizhi drug pair on key targets for the treatment of ovarian cancer.

The results of KEGG enrichment analysis pathway were shown in Table 2. Pathways in cancer, hepatitis B, endocrine resistance, proteoglycans in cancer, AGE-RAGE signaling pathway in diabetes complications, PI3K-Akt signaling pathway, apoptosis, platinum drug resistance, TNF signaling pathway, IL-17 signaling pathway, progesterone-mediated oocyte maturation, and the prolactin signaling pathway were included in Fig. 5.

**Table 2:** Pathway enrichment analysis of Danshen-Guizhi drug pair on key targets for the treatment of ovarian cancer.

GO	Description	LogP	Count	Enrichment
hsa05200	Pathways in cancer	−66.229 27	48	35.521 46
hsa05161	Hepatitis B	−44.160 11	28	67.430 58
hsa05169	Epstein-Barr virus infection	−33.737 93	26	37.065 88
hsa01522	Endocrine resistance	−33.702 80	20	88.803 66
hsa05167	Kaposi sarcoma-associated herpesvirus infection	−32.865 27	23	50.535 69
hsa05163	Human cytomegalovirus infection−	−32.508 07	24	42.625 76
hsa05166	Human T-cell leukemia virus 1 infection	−32.079 13	25	36.123 52
ko04933	AGE-RAGE signaling pathway in diabetic complications	−31.234 36	19	81.807 01
hsa05205	Proteoglycans in cancer	−29.776 68	22	43.016 82
hsa05215	Prostate cancer	−28.844 49	18	75.221 93
hsa04151	PI3K-Akt signaling pathway	−27.611 97	24	26.921 53
hsa04210	Apoptosis	−27.498 32	19	53.635 06
hsa01524	Platinum drug resistance	−27.252 35	16	93.426 32
hsa05222	Small cell lung cancer	−26.999 62	17	73.195 75
ko05166	HTLV-I infection	−26.430 33	21	34.966 44
hsa05212	Pancreatic cancer	−26.360 33	16	83.172 21
hsa04668	TNF signaling pathway	−25.812 31	17	63.011 99
ko04657	IL-17 signaling pathway	−25.408 17	16	73.334 64
hsa04914	Progesterone-mediated oocyte maturation	−13.307 95	10	41.384 23
ko04917	Prolactin signaling pathway	−13.149 51	9	54.804 54

### Construction of “component-target-pathway” network of Danshen-Guizhi drug pair

**Figure 6:**
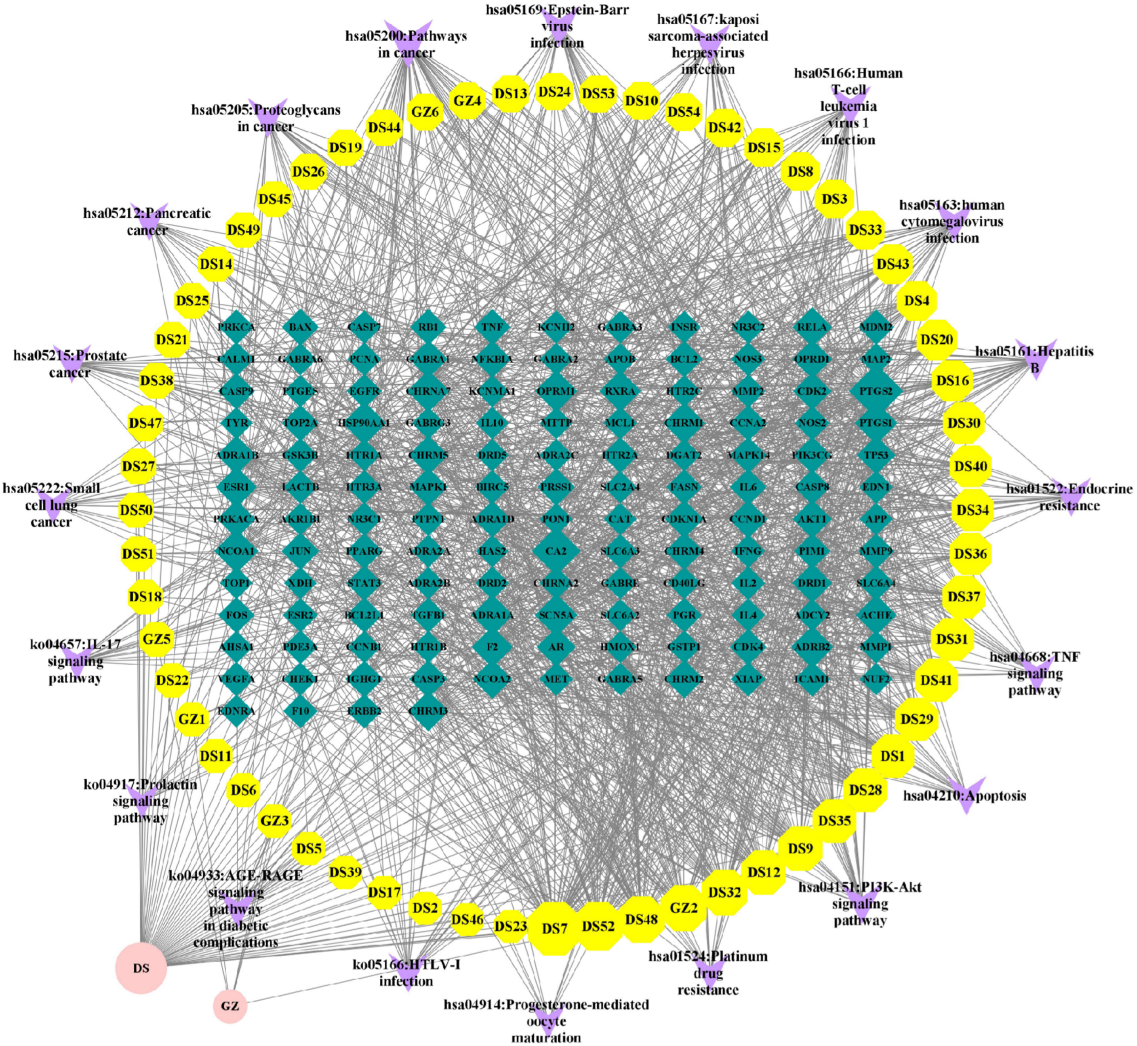
Network diagram of “component-target-pathway”. The circle represents the drug, the octagon represents the drug component, the quadrilateral represents the target, and the inverted triangle represents the pathway. The size of the node is related to the degree value; the greater the degree value, the larger the node.

The results of effective active components, potential targets and enrichment pathways of the Danshen-Guizhi drug pair were analyzed by Cytoscape3.8.0 built-in plug-in CytoNCA, the core components and core targets of Danshen-Guizhi drug pair in the treatment of ovarian cancer were obtained, as shown in Fig. 6.

Cytoscape analysis results showed that the active ingredients DS7 luteolin, DS52 tanshinone IIA, DS48 salviolone, GZ2 beta-sitosterol, DS32 dihydrotanshinone lactone, DS28 cryptotanshinone and so on, among which the highest degree of luteolin was 58, the betweenness centrality was 0.204, and the closeness centrality was 0.476. It can be judged that luteolin was the core component of the Danshen-Guizhi drug pair in the treatment of ovarian cancer, followed by tanshinone (connectivity was 46, betweenness centrality was 0.048, and closeness centrality was 0.422), salviolone (connectivity was 39, betweenness centrality was 0.099, and closeness centrality was 0.414), and β-sitosterol (connectivity is 38, betweenness centrality was 0.083, closeness centrality was 0.431), which can be considered to be effective core components in the treatment of ovarian cancer.

The core target nodes were sorted by degree value (connectivity): PTGS2 (connectivity 61), CA2 (connectivity 46), NCOA1 (connectivity 33), HSP90AA1 (connectivity 31), ADRB2 (connectivity 29), SCN5A (connectivity 29), PTGS1 (connectivity 29), CHRNA7 (connectivity 28), OPRM1 (connectivity 28), CHRM1 (connectivity 27), RXRA (connectivity 26), ESR1 (connectivity 25), F2 (connectivity 25), ADRA1A (connectivity 25), ACHE (connectivity 24), CHRM3 (connectivity 23), RELA (connectivity 21), DRD1 (connectivity 21), AR (connectivity 20), and NCOA2 (connectivity 20). According to the degree value, the above targets can be judged as effective core targets for the treatment of ovarian cancer, and PTGS2 is the main core target.

### Molecular docking analysis

**Figure 7:**
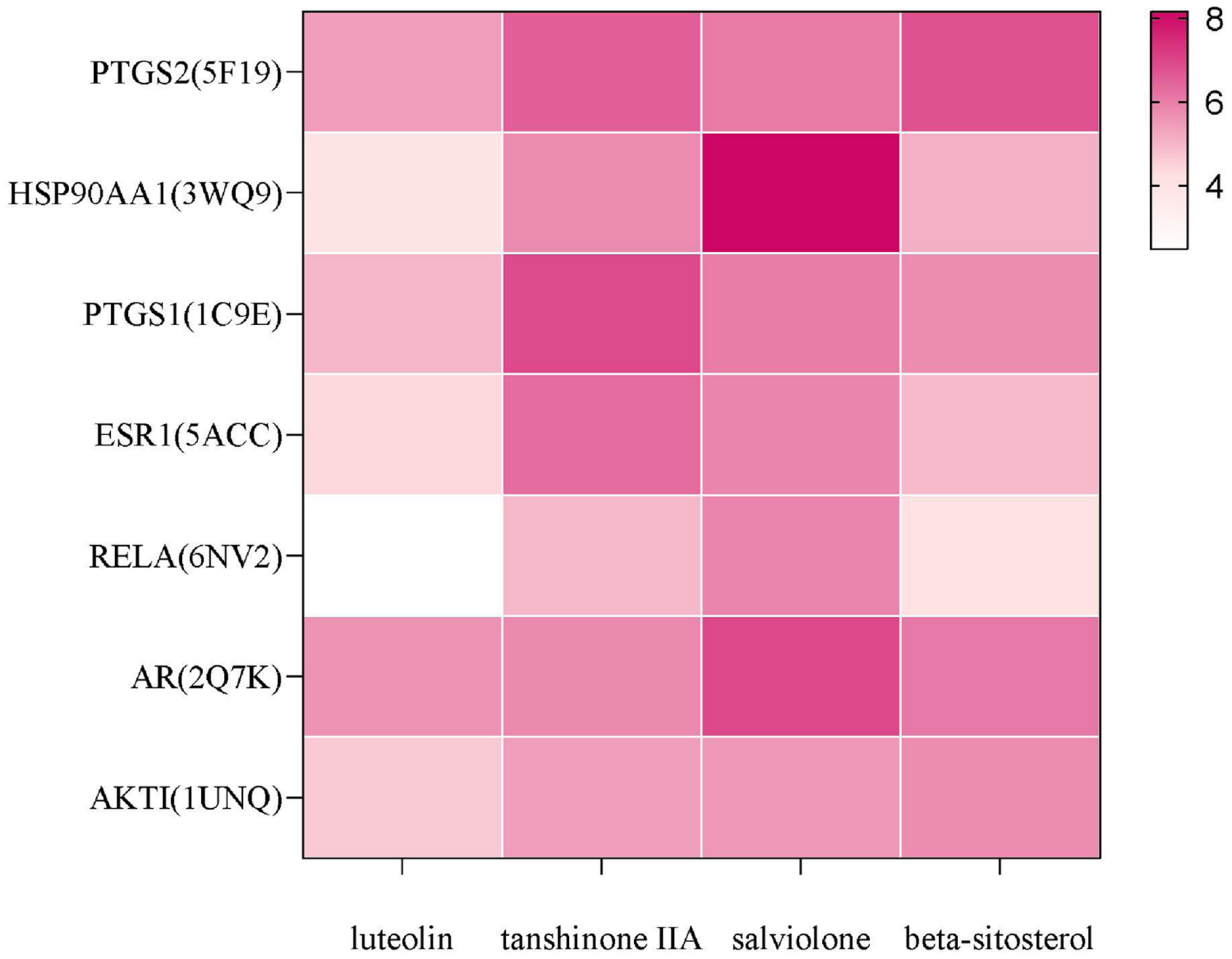
Docking results of main components with core targets.

In AutoDock docking, the score of the binding ability between active ingredients and core targets of the Danshen-Guizhi drug pair was greater than 4.25, indicating that there was a binding ability between active ingredients and targets, greater than 5.25, indicating that the molecule has a good relationship with the target, and greater than 7.0, indicating that the molecule has an excellent binding ability to the target (Hsin, Ghosh & Kitano, 2013). The results of molecular docking analysis showed that the docking scores of salviolone and tanshinone IIA with the core targets were higher, and the docking scores of PTGS2, AR and PTGS1 with the active components were higher. In the total docking scores, 89% of the total docking scores were higher than 4.25, and 68% of the total docking scores were higher than 5.25, indicating that the predicted targets had good binding ability with the active components. The results of molecular docking were shown in Fig. 7. The higher the score was, the deeper the color was. The highest value was 8.16.

### Inhibition of core components on ovarian cancer cells

**Figure 8:**
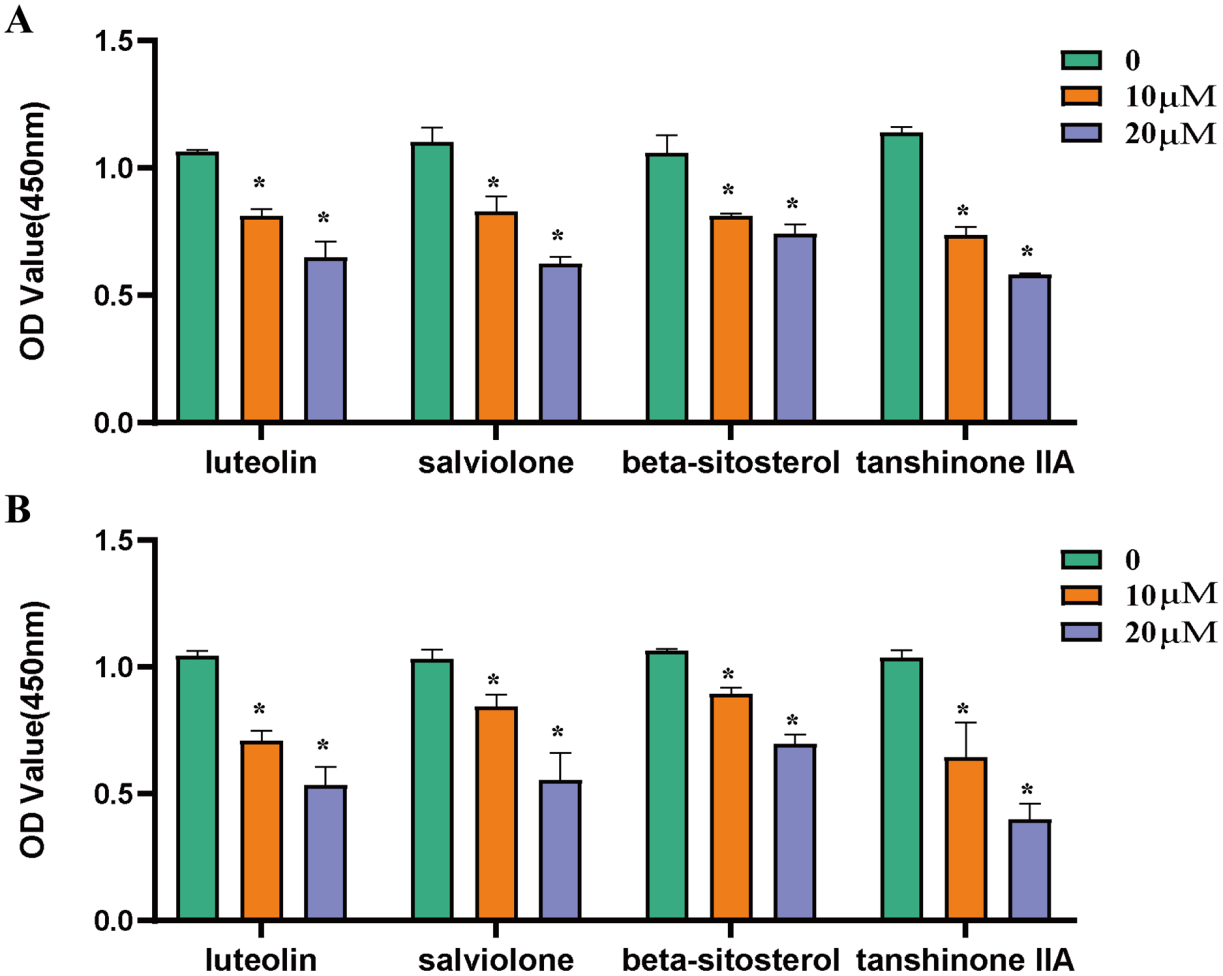
Inhibition of core components on ovarian cancer cells. (A) The inhibitory effects of luteolin, salviolone, β-sitosterol and tanshinone IIA at different concentrations (0,10 μm, 20 μm) on SKOV-3 cells. (B) The inhibitory effects of luteolin, salviolone, β-sitosterol and tanshinone IIA at different concentrations (0,10 μm, 20 μm) on A2780 cells. *P < 0.05, statistically significant.

The effects of luteolin, salviolone, β-sitosterol as well as tanshinone IIA on SKOV3 and A2780 cells were determined by MTS assay. Compared with the control group, the activity of SKOV3 cells (Fig. 8A) and A2780 cells (Fig. 8B) were significantly inhibited by 10 μm, 20 μm luteolin, salviolone, β-sitosterol as well as tanshinone IIA, as shown in Fig. 8.

### Expression of PTGS2 mRNA and PGE2

**Figure 9:**
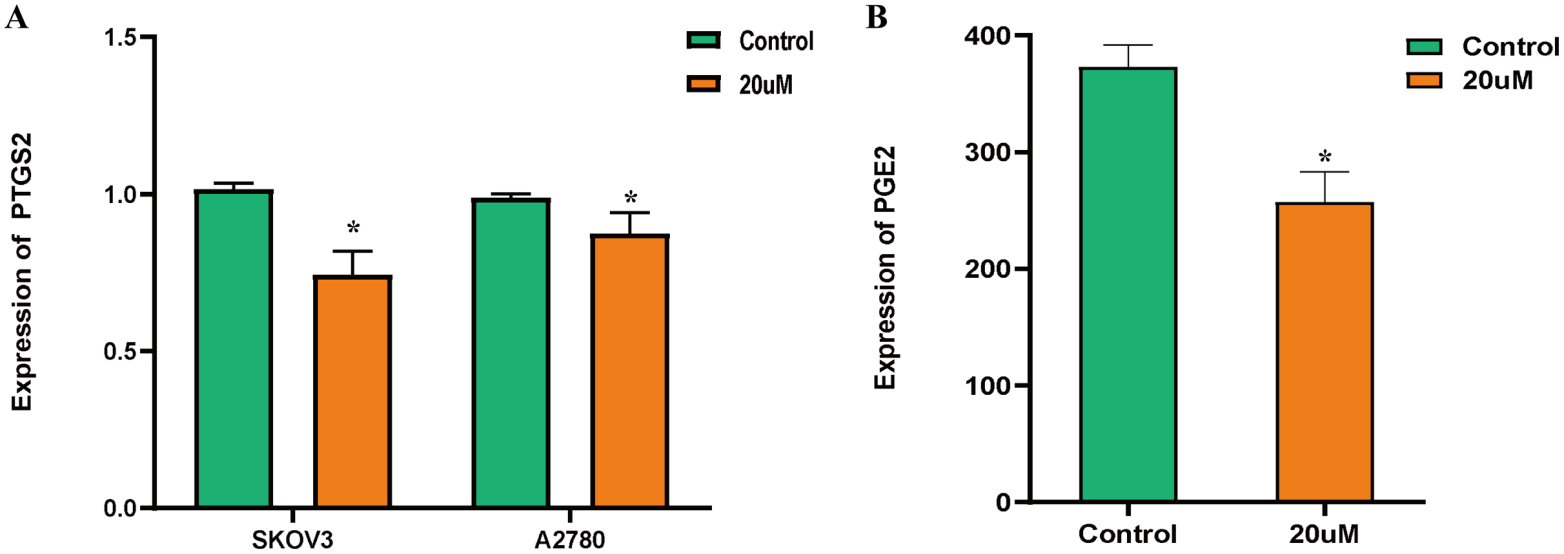
Expression of PTGS2 mRNA and PGE2. (A) qRT-PCR detection of PTGS2 mRNA expression. Compared with the control group, the expression of PTGS2 was significantly inhibited by 20 μm salviolone in SKOV3 and A2780 cells; (B) Detection of PGE2 expression by ELISA. Compared with the control group, the expression of PGE2 was significantly down-regulated by 20 μm Salviolone. *P < 0.05, statistically significant.

The expression of PTGS2 mRNA was evaluated by qRT-PCR. The expression of PTGS2 in SKOV3 and A2780 cells were significantly inhibited by salviolone (Fig. 9A). The expression of PGE2 was determined by ELISA. The expression of PGE2 in SKOV3 cells was significantly down-regulated by salviolone (Fig. 9B).

## Discussion

This was the first study to explore the main effective components and potential mechanism of the Danshen-Guizhi drug pair in the treatment of ovarian cancer by network pharmacology. The “component-target-pathway” network of the Danshen-Guizhi drug pair was constructed to analyze the effective active components, important targets and potential mechanisms of the Danshen-Guizhi drug pair. Finally, the molecular docking and *in vitro* study were used to verify the network.

Four important active components were screened from the network of “component-target-pathway” of the Danshen-Guizhi drug pair, which were luteolin, salviolone, β-sitosterol and tanshinone IIA. Luteolin is a polyphenol flavonoids. Luteolin has anti-inflammatory, antiallergic, antioxidant and immuneregulatory pharmacological activities that can be used as antioxidants or prooxidants (Lin et al., 2008; Lopez-Lazaro, 2009; Wang et al., 2018). At the same time, luteolin has a strong role in cell apoptosis, cell cycle regulation, proliferation and differentiation of cancer cells (Tavsan & Kayali, 2019). Salviolone is an antioxidant component extracted from rosemary. Salviolone has many pharmacological effects such as anti-radiation, anti-oxidation, anti-cancer, and inhibiting the migration and invasion of cancer cells (Zhang, Li & Ji, 2018). Beta-sitosterol, a plant derived nutrient, studies have shown that β-sitosterol can interfere with a variety of cell signaling pathways, including apoptosis, cell proliferation and angiogenesis, metastasis and inflammation (Bin Sayeed & Ameen, 2015). Tanshinone IIA is an active component extracted from Danshen. Tanshinone IIA can induce apoptosis of ovarian cancer TOV-21G cells by up-regulating miR-205, which mainly reveals the potential therapeutic application of tanshinone IIA in ovarian cancer (Li et al., 2018). *In vitro* experiments showed that the activity of SKOV3 and A2780 cells were significantly inhibited by luteolin, salviolone, β-sitosterol and tanshinone IIA. This indicated that the Danshen-Guizhi drug pair might treat ovarian cancer through these components.

By analyzing the “component-target-pathway” network of the Danshen-Guizhi drug pair, the main selected targets were PTGS2, HSP90AA1, PTGS1, AKT1 and ESR1. PTGS2 enhanced the proliferation and migration of human ovarian cancer CAOV-3 cells mainly by activating phosphatidylinositol 3 kinase/protein kinase B (PI3-k/Akt) pathway (Gu et al., 2008). It was reported that HSP90AA1 could affect the survival of tumor cells, the invasion and migration of cancer cells, and was closely related to the poor prognosis of tumours. HSP90AA1 RNAi could inhibit the proliferation of ovarian cancer SKOV3 cells and increase its apoptosis (Calderwood et al., 2006; Chu et al., 2013). AKT1 (AKT) is a serine/threonine-specific protein kinase. The activation of PI3K/AKT target caused about 70% of ovarian cancer cells to be activated, which maked many signaling pathways related to cell growth, proliferation, survival, metabolism and angiogenesis overactive (Li, Zeng & Shen, 2014). ESR1 is an important component of estrogen receptor. It is generally believed that ESR1 regulated the expression of protein-coding genes closely related to cell proliferation, survival, differentiation, and participation in the process of cancer (Hu, Chen & Ding, 2018). Therefore, it can be speculated that the Danshen-Guizhi drug pair mainly plays biological functions in the treatment of ovarian cancer through the above targets.

Based on the enrichment results and the analysis of the Danshen-Guizhi drug pair “component-target-pathway” network, it can be considered that the main biological processes are apoptosis, cell proliferation and tumor immunity. The main active components involved were luteolin, salviolone, β-sitosterol and tanshinone IIA.

The pathways involved in apoptosis are mainly apoptosis pathway and PI3K-Akt signaling pathway. PI3K-Akt signal transduction is usually destroyed in human cancer, and AKT1 is an important part of this pathway, which affects multiple processes directly involved in tumorigenesis (Brown & Banerji, 2017). AKT1, CCND1, EGFR, HSP90AA1, MAPK1, RELA and TP53 are involved in the PI3K-Akt signal pathway. The main pathways involved in cell proliferation are PI3K-Akt signaling pathway and proteoglycan pathway in cancer. As an important macromolecule, proteoglycans can participate in the biological process of various cancers and plays an important role. It can affect the progression of tumors by affecting the proliferation, angiogenesis and metastasis of tumor cells (Goldoni et al., 2008). AKT1, CCND1, CASP3, CDKN1A, ESR1, MAPK1, TP53 and other proteins participate in the proteoglycan pathway in cancer. The main pathway involved in tumor immunity is IL-17 signaling pathway. IL-17A is a multi-effect cytokine, and its expression is related to the pathological differentiation level and lymph node metastasis of ovarian cancer patients (Wang et al., 2020). CASP3, HSP90AA1, MAPK1, PTGS2, MAPK1 and TP53 are involved in this pathway.

PTGS2 and PGE2 could promote cancer progression by affecting the differentiation and survival of tumor-associated immune cells, and shifting the tumor microenvironment into an immunosuppressive environment (Wang & Dubois, 2010). PTGS2 and PGE2 inhibition may be useful to augment cancer immunotherapy (Chen & Smyth, 2011). In our study, *in vitro* cell experiments showed that the expression of PTGS2 in SKOV3 cells as well as A2780 cells and PGE2 in SKOV3 cells were significantly inhibited by salviolone. This further indicates the mechanism of the Danshen-Guizhi drug pair in the treatment of ovarian cancer may be inhibiting the expression of PTGS2.

## Conclusion

In summary, the effective components of the Danshen-Guizhi drug pair in the treatment of ovarian cancer may be salviolone, luteolin, β-sitosterol and tanshinone IIA. The most effective target is PTGS2. The potential mechanism of the Danshen-Guizhi drug pair in the treatment of ovarian cancer may be regulating the proliferation, apoptosis and tumor immunity. This can provide a theoretical basis for the future application of the Danshen-Guizhi drug pair in the treatment of ovarian cancer, and also provide a reference for the clinical application of the Danshen-Guizhi drug pair. Our results need to be validated by further researches.

## Supplemental Information

10.7717/peerj.13148/supp-1 Supplemental Information 1 Raw data for OD & protein expression.

10.7717/peerj.13148/supp-2 Supplemental Information 2 Ovarian cancer-related targets.

## Additional Information and Declarations

### Data Availability

The following information was supplied regarding data availability: The raw measurements are available in the Supplemental File.
